# Characterisation of the volatile profile of microalgae and cyanobacteria using solid-phase microextraction followed by gas chromatography coupled to mass spectrometry

**DOI:** 10.1038/s41598-022-07677-4

**Published:** 2022-03-07

**Authors:** Lara Moran, Gemma Bou, Noelia Aldai, Martina Ciardi, Ainoa Morillas-España, Ana Sánchez-Zurano, Luis Javier R. Barron, Tomas Lafarga

**Affiliations:** 1 Lactiker Research Group, Department of Pharmacy and Food Science, University of the Basque Country (UPV/EHU), 01006 Vitoria-Gasteiz, Spain; 2 Department of Chemical Engineering, University of Almería, CIESOL Solar Energy Research Centre, Joint Centre University of Almeria-CIEMAT, 04120 Almería, Spain

**Keywords:** Biochemistry, Analytical biochemistry, Mass spectrometry

## Abstract

Microalgae and microalgae-derived ingredients are one of the top trends in the food industry. However, consumers’ acceptance and purchase intention of a product will be largely affected by odour and flavour. Surprisingly, the scientific literature present a very limited number of studies on the volatile composition of microalgae and cyanobacteria. In order to fill the gap, the main objective of the present study was to elucidate the volatile composition of seven microalgal and cyanobacterial strains from marine and freshwaters, with interest for the food industry while establishing its potential impact in odour. Among the seven selected strains, *Arthrospira platensis* showed the highest abundance and chemical diversity of volatile organic compounds (VOCs). Aldehydes, ketones, and alcohols were the families with the highest diversity of individual compounds, except in *Arthrospira platensis* and *Scenedesmus almeriensis* that showed a profile dominated by branched hydrocarbons. Marine strains presented a higher abundance of sulfur compounds than freshwater strains, while the ketones individual profile seemed to be more related to the taxonomical domain. The results of this study indicate that the VOCs composition is mainly driven by the individual strain although some volatile profile characteristics could be influenced by both environmental and taxonomical factors.

## Introduction

Microalgae and cyanobacteria are being mass cultured for a wide range of industrial applications, however, most of the microalgal biomass is currently being used for the manufacture of food supplements (rich in protein, carotenoids, or polyunsaturated fatty acids)^1^. *Arthrospira maxima* and *Arthrospira platensis* (*AP*) are the most common strains of cyanobacteria used in food applications (commercialised as Spirulina). The microalga *Chlorella vulgaris* (*CV*) is also widely used in the food industry, mainly in Asia^2^. Other species from the genus *Nannochloropsis*, *Scenedesmus*, *Haematococcus*, *Dunaliella,* or *Tetraselmis* have also been used for the manufacture of food supplements at lab-scale^1^. In addition, the microalga *Tetraselmis chuii* has been authorised as a novel food in accordance with Regulation (EC) No 258/97^3^.

Microalgae and microalgae-derived ingredients are one of the top trends in the food industry, and the amount of microalgae-containing foods launched into the marked has steadily increased during the last decade, being this trend likely to continue to grow^1,4^. Among the main constrains related to the use of microalgae in food industry, there are some related with their production such as high production costs and low yields^5^, but also others related with quality such as their (generally) green colour and strong marine taste and odour^6,7^. Odour and flavour are of key importance as both will largely affect the consumers’ acceptance or rejection of a product as well as the purchase intention. In the scientific literature, there is a very limited number of studies on the volatile composition of microalgae and cyanobacteria, and to the best of the authors’ knowledge, there are no studies on the relationship between volatile profile and their origin (marine or freshwater). Among the few studies on the matter, sulfur compounds, esters, and alcohols have been identified in the biomass of *Crypthecodinium cohnii* while aldehydes and alcohols were detected in *Schizochytrium limacinum*^8^. Several volatile compounds identified in seafood such as sulfur compounds (dimethyl disulphide, dimethyl trisulphide, and methional), diketones (2,3-butanedione, 2,3-pentadione and 2,3-octanedione), and terpenoids (α- and β-ionone) were identified in different microalgal strains namely *Botryococcus braunii*, *Rhodomonas*, *Chlorella*, *Tetraselmis*, and *Nannochloropsis*^8,9^.

The aim of the present study is to determine the volatile composition and potential odour impact of seven strains of microalgae and cyanobacteria from marine and freshwater analysed by solid-phase microextraction followed by gas chromatography coupled to mass spectrometry.

## Results and discussion

Identification and semi-quantitative information of the individual VOCs found in the microalgal and cyanobacterial strains are listed in Table 1. Results listed by chemical family are presented in Table 2.

**Table 1:** Relative abundance (arbitrary area units × 10^3^) of volatile compounds in *Isochrysis galbana (IG)*, *Nannochloropsis gaditana (NG), Tetraselmis* sp. *(TS), Scenedesmus almeriensis (SA)*, *Chlorella vulgaris (CV) Synechococcus* sp. (*SY*) and *Arthrospira platensis* (*AP*) classified by domain (eukaryote-microalgae and prokaryote-cyanobacteria) and environment (marine and freshwater).

LRI	CAS number	*m/z* ions*	Compound		Prokaryote						
					Marine			Freshwater		Marine	Freshwater
					*IG*	*NG*	*TS*	*SA*	*CV*	*SY*	*AP*
**Acyclic aldehydes**					863^a^	15.2^c^	60.0^b,c^	36.1^b,c^	162^b^	48.7^b,c^	323^b^
790	123-38-6	58,29,28,27	Propanal	^P^	54.3^a^	1.01^b^	1.49^b^	4.54^b^	12.5^a^	ND	ND
811	78-84-2	43,41,72,39	Propanal, 2-methyl-	^P^	ND	ND	2.29^b^	ND	ND	ND	308^a^
872	123-72-8	44,43,72,41	Butanal	^P^	6.87	ND	0.835	ND	1.89	ND	ND
916	590-86-3	44,43,41,58	Butanal, 3-methyl-	^P^	21.7	ND	11.8	9.30	9.18	ND	ND
978	110-62-3	44,58,29,41	Pentanal		23.7^a^	ND	3.78^b^	3.42^b^	14.2^a^	2.69^b^	ND
1081	66-25-1	44,56,41,43	Hexanal	^P^	142^a^	9.82^b^	28.9^b^	ND	72.7^a^	3.87^b^	ND
1093	1115-11-3	55,84,29,27	2-Butenal, 2-methyl-		136^a^	1.06^c^	5.84^b,c^	10.8^b^	6.55^b,c^	ND	ND
1131	1576-87-0	55,84,83,41	2-Pentenal, (*E*)-		12.9^a^	0.380^b^	0.232^b^	ND	5.24^a,b^	0.654^b^	7.78^a^
1150	19780-25-7	41,39,98,55	2-Butenal, 2-ethyl-		65.2^a^	ND	0.508^b^	1.07^b^	1.36^b^	0.100^b^	ND
1159	623-36-9	41,98,69,39	2-Pentenal, 2-methyl-	^P^	254^a^	1.23^b^	1.59^b^	4.56^b^	7.73^b^	ND	ND
1184	111-71-7	70,41,44,43	Heptanal	^P^	24.1^a^	1.12^b^	1.83^b^	ND	3.11^b^	0.897^b^	6.68^b^
1208	28467-88-1	55,41,112,39	2-Hexenal, 2-methyl-		13.4	ND	ND	ND	ND	ND	ND
1220	6728-26-3	41,42,39,83	2-Hexenal, (*E*)-	^P^	7.84^a^	ND	0.618^b^	ND	1.68^b^	ND	ND
1241	6728-31-0	41,68,29,55	4-heptenal		19.3^a^	ND	ND	ND	1.29^b^	ND	ND
1254	3491-57-4	55,41,43,27	2-Pentenal, 2-ethyl-		32.7^a^	ND	ND	ND	2.10^b^	ND	ND
1473	4313-02-4	81	2,4-Heptadienal, (*E,Z*)-	^P^	3.75	ND	ND	ND	3.32	ND	ND
1492	2548-87-0	107,122,77	2-Octenal		9.06^a^	0.599^b^	0.309^b^	ND	3.89^b^	ND	ND
1504	4313-03-5	81,110,41,53	2,4-Heptadienal, (*E,E*)-	^P^	35.7^a^	ND	ND	2.41^b^	13.4^a,b^	40.4^a^	ND
**Cyclic aldehydes**					40.2^a^	1.09^b,c^	21.9^a,b^	25.0^a,b^	6.78^b,c^	0.838^c^	44.8^a^
1539	100-52-7	77,106,105	Benzaldehyde	^p^	36.9^a^	1.09^b,c^	12.8^a,b^	12.9^a,b^	5.34^b,c^	0.838^c^	10.6^a,b^
1638	432-25-7	41,137,152	β-Cyclocitral^1^	^P^	3.25^b^	ND	5.32^b^	4.58^b^	1.03^b^	ND	21.0^b^
1661	116-26-7	107,91,121	Safranal^2^	^P^	ND	ND	3.79^a,b^	7.56^a^	0.410^b^	ND	13.3^a^
**Aromatic hydrocarbons**					132^b^	47.9^d^	83.2^c,d^	231^b^	32.5^d^	185^b,c^	679^a^
1037	108-88-3	91,92,65,39	Benzene, methyl-	^P^	81.8^b,c^	35.8^c^	47.4^c^	135^a,b^	11.0^c^	146^a,b^	452^a^
1124	100-41-4	91,106,51,65	Benzene, ethyl-	^P^	5.96^a^	1.15^b,c^	1.73^b^	0.493^c^	1.55^b,c^	4.29^a^	ND
1258	100-42-5	104,103,78	Benzene, ethenyl-	^P^	42.6^a^	10.9^b^	34.0^a^	ND	18.5^b^	35.1^a^	ND
1428	1014-60-4	175,57,41,29	Benzene, 1,3-bis(1,1-dimethylethyl)-		1.08^b^	ND	ND	95.9^a^	1.47^b^	ND	227^a^
**Branched hydrocarbons**					120^c^	14.6^d^	12.5^d^	888^b^	130^c^	67.8^c,d^	2496^a^
715	592-27-8	43,57,42,41	Heptane, 2-methyl-		ND	ND	ND	7.53^a^	0.612^b^	7.50^a^	11.5^a^
722	589-53-7	43,70,71,41	Heptane, 4-methyl-		ND	ND	ND	3.60	ND	10.0	4.45
808	2213-23-2	43,85,57,71,	Heptane, 2,4-dimethyl-	^P^	ND	ND	ND	76.8^a^	ND	12.1^b^	142^a^
846	3074-71-3	43,84,85,57	Heptane, 2,3-dimethyl-	^P^	ND	ND	ND	3.18	ND	3.67	11.0
851	2216-34-4	43,85,71,41	Octane, 4-methyl-		ND	ND	ND	58.5^a^	ND	22.2^b^	150^a^
915	1603-01-6	81,67,68,95	1,4-Heptadiene, 3-methyl-		ND	ND	ND	7.46	ND	ND	18.0
955	14676-29-0	57,41,98,43	Heptane, 3-ethyl-2-methyl-		ND	ND	ND	85.5^b^	ND	ND	266^a^
965	15869-86-0	57,43,41,71	Octane, 4-ethyl-		ND	ND	ND	2.62^b^	ND	ND	8.62^a^
996	5911-04-6	57,71,43,56	Nonane, 3-methyl-		ND	ND	ND	11.1^b^	ND	ND	37.5^a^
1007	17302-27-1	57,43,85,41	Nonane, 2,5-dimethyl-		ND	4.35^c^	ND	38.4^b^	ND	ND	126^a^
1010		43,57,85,71	Unknown		ND	ND	ND	77.9^a^	1.47^b^	ND	253^a^
1031	62016-19-7	43,71,57,85	Octane, 6-ethyl-2-methyl-		ND	ND	ND	4.44	ND	ND	ND
1033		71,57,43,85	Unknown		ND	ND	ND	4.24^b^	114^a^	ND	36.7^b^
1039		41,85,71,57	Unknown		ND	ND	ND	112^b^	11.0	ND	339^a^
1043	62016-18-6	43,57,71,85	Octane, 5-ethyl-2-methyl-		ND	ND	ND	48.9^b^	ND	ND	148^a^
1051	13151-35-4	57,43,85,41	Decane, 5-methyl-		ND	ND	ND	29.2^b^	ND	ND	88.6^a^
1054	2847-72-5	43,71,57,41	Decane, 4-methyl-		ND	ND	ND	36.9^b^	ND	ND	107^a^
1081	13151-34-3	57,71,43,41	Decane, 3-methyl-		ND	ND	ND	9.36^b^	ND	ND	70.0^a^
1088		43,57,71,85	Unknown		ND	ND	ND	59.5^b^	ND	ND	181^a^
1093	17302-23-7	43,57,71,85	Nonane, 4,5-dimethyl-		ND	ND	ND	18.1^a,b^	ND	7.26^b^	49.9^a^
1102	52670-34-5	43,71,57,41	Octane, 2,3,6,7-tetramethyl-		ND	ND	ND	21.0	ND	ND	11.3
1102	1632-70-8	43,57,71,85	Undecane, 5-methyl-		ND	ND	ND	2.79^b^	ND	ND	8.96^a^
1104	2980-69-0	43,71,57,41	Undecane,4-methyl-		ND	ND	ND	1.13	ND	5.12	3.68
1115		43,57,71,85	Unknown		ND	ND	ND	2.70	ND	ND	8.26
1157	7045-71-8	43,57,71,85	Undecane, 2-methyl		ND	ND	ND	11.1	ND	ND	44.0
1175		57,43,85,71	Unknown		ND	ND	ND	8.82^b^	ND	ND	24.6^a^
1195	17312-80-0	43,85,57,41	Undecane, 2,4-dimethyl-		ND	ND	ND	3.32^b^	ND	ND	9.61^a^
1198	17301-23-4	57,71,43,41	Undecane, 2,6-dimethyl-		ND	ND	ND	19.1^b^	ND	ND	65.8^a^
1209	17301-33-6	43,71,57,41	Undecane, 4,8-dimethyl-		ND	ND	ND	6.94	ND	ND	20.0
1218	17301-32-5	43,57,71,85	Undecane, 4,7-dimethyl-		ND	ND	ND	9.55^b^	ND	ND	24.8^a^
1225		57,43,71,85	Unknown		ND	ND	ND	4.16^b^	ND	ND	12.5^a^
1232	17312-82-2	57,43,71,41	Undecane, 4,6-dimethyl-		ND	ND	ND	6.89	ND	ND	18.3
1241		57,71,85,99	Unknown		ND	ND	ND	3.04	ND	ND	5.59
1244		43,57,55,41	Unknown		ND	ND	ND	7.80^b^	ND	ND	30.2^a^
1247		71,43,71,85	Unknown		ND	ND	ND	41.2	ND	ND	116
1255		57,43,71,85	Unknown		ND	ND	ND	8.85	ND	ND	19.4
1261		57,43,85,71	Unknown		ND	ND	ND	3.40	ND	ND	9.58
1277	61141-72-8	57,43,71,41	Dodecane, 4,6-dimethyl-		ND	ND	ND	1.92	ND	ND	8.57
1395		69,41,111,55	2-Hexene, 4-ethyl-2,3-dimethyl-		1.63	ND	1.47	4.36	ND	ND	4.19
1477	26456-76-8	57,70,41,29	2-Hexene, 3,5,5-trimethyl-		118^a^	10.2^b^	11.0^b^	24.3^a,b^	2.56^b^	ND	2.39^b^
**Alicyclic hydrocarbons**					40.4^a^	1.16^c^	5.75^b,c^	11.5^a,b^	4.14^b^	21.6^a^	28.3^a^
742	108-87-2	83,55,41,98	Cyclohexane, methyl-	^P^	ND	ND	ND	ND	ND	3.32	ND
833	6876-23-9	55,97,41,56	Cyclohexane, 1,2-dimethyl	^P^	ND	ND	ND	0.932^b^	ND	3.65^a^	ND
879	1678-91-7	83,55,82,41	Cyclohexane, ethyl-	^P^	ND	ND	0.239	4.84	ND	6.68	9.34
931	65378-76-9	109,67,81,124	1-Cyclopentene, 1,2,4,4-tetramethyl-		37.1^a^	1.16^c^	3.22^a,b,c^	1.14^c^	3.63^a,b,c^	7.96^a,b^	14.0^a,b^
1459	17299-41-1	96,95,110,67	2-Cyclohexen-1-one, 3,4,4-trimethyl-		3.30	ND	2.29	4.62	0.506	ND	5.01
**Linear hydrocarbons**					143^b^	33.9^c^	29.6^c^	141^b^	42.5^c^	170^b^	861^a^
607	504-60-9	67,68,53,39	1,3-Pentadiene		4.13	0.655	ND	ND	0.593	ND	ND
629	142-82-5	43,41,29,57	Heptane	^P^	71.4	31.9	28.4	46.3	38.4	56.8	145
801	111-65-9	43,41,57,29	Octane	^P^	51.6	ND	0.851	9.07	2.36	13.9	23.6
900	111-84-2	43,57,41,85	Nonane	^P^	ND	ND	ND	8.03^b^	ND	ND	35.2^a^
927	63216-69-3	39,41,27,67	2,5-Octadiene		15.8	0.673	0.0121	ND	ND	ND	ND
1000	124-18-5	57,43,41,71	Decane	^P^	ND	ND	0.388^b^	64.5^b^	1.17^b^	ND	217^a^
1098	1120-21-4	57,43,71,41	Undecane	^P^	ND	ND	ND	5.61	ND	ND	16.2
1099	22038-68-2	79,77,93,91	1,3,6-Octatriene		ND	0.706	ND	ND	ND	ND	ND
1296	629-50-5	57,43,71,41	Tridecane	^P^	ND	ND	ND	7.91^b^	ND	58.2^a^	29.0^a^
1500	629-62-9	57,43,71,85	Pentadecane	^P^	ND	ND	ND	ND	ND	36.5	107
1595	544-76-3	57,43,71,85	Hexadecane	^P^	ND	ND	ND	ND	ND	1.18	34.7
1696	629-78-7	57,43,71,85	Heptadecane	^P^	ND	ND	ND	ND	ND	3.79^b^	252^a^
**Acyclic alcohols**					533^a^	102^b^	66.5^b,c^	138^a,b^	117^b,c^	4.93^c^	372^a,b^
1140	71-36-3	56,41,43,41	1-Butanol	^P^	ND	ND	ND	ND	ND	2.89	ND
1154	616-25-1	57,41,39,43	1-Penten-3-ol	^P^	313^a^	69.0^a^	20.9^b^	110^a^	64.6^a^	1.21^b^	7.10^b^
1199	598-75-4	55,42,43,41	2-Butanol, 3-methyl-	^P^	ND	ND	1.56	ND	2.96	ND	ND
1247	71-41-0	42,55,41,70	1-Pentanol	^P^	15.6^a^	0.0771^c^	3.89^b^	ND	2.21^b^	ND	30.6^a^
1304	1576-96-1	57,41,39,44	2-Penten-1-ol, (*E*)-	^P^	24.4	2.21	ND	2.92	ND	ND	ND
1313	1576-95-0	57,68,41,44	2-Penten-1-ol, (*Z*)-	^P^	57.2^a^	15.6^a,b^	3.48^b^	11.6^a,b^	6.56^b^	ND	ND
1347	111-27-3	56,55,43,41	1-Hexanol	^p^	7.13^b^	1.24^b^	2.69^b^	ND	2.13^b^	ND	149^a^
1398	928-94-9	57,41,82,67	2-Hexen-1-ol	^P^	17.4^b^	6.65^b,c^	6.21^b,c^	4.51^b,c^	3.94^b,c^	0.825^c^	119^a^
1442	3391-86-4	57,43,72,41	1-Octen-3-ol	^P^	86.1^a^	6.53^b^	27.8^a,b^	8.54^b^	34.2^a,b^	ND	65.8^a^
1787	81912-03-0	57,83,84,41	1,3-Heptadien-5-ol, 6,6-dimethyl-		5.72	ND	ND	ND	ND	ND	ND
1887	17920-92-2	43,41,69,39	1,7-Nonadien-4-ol, 4,8-dimethyl-		6.82	0.450	0.073	0.320	ND	ND	0.06
**Cyclic alcohols**					50.4^a^	7.12^b^	44.4^a^	62.3^a^	15.3^b^	2.06^b^	201^a^
1420		135,150,91	Unknown		4.46^b^	0.735^b^	0.371^b^	1.35^b^	0.893^b^	2.06^b^	39.5^a^
1613	69542-91-2	95,43,57,41	Cyclohexanol, 2,4-dimethyl-		45.9^a^	6.38^b^	44.0^a^	60.9^a^	14.4^b^	ND	161^a^
**Acyclic ketones**					725^a^	35.8^c^	31.7^c^	77.7^b^	102^b^	78.7^b^	419^b^
815	67-64-1	43,58,15,42	2-Propanone	^P^	36.9^a,b^	7.57^c^	3.21^c^	ND	7.77^c^	30.3^a,b^	211^a^
902	78-93-3	43,72,29,27	2-Butanone	^P^	38.2^a^	2.52^b^	3.42^b^	ND	5.13^b^	8.32^b^	ND
976	107-87-9	43,86,41,58	2-Pentanone	^P^	80.1^a^	2.73^b^	1.63^b^	2.72^b^	5.45^b^	ND	3.44^b^
1020	1629-58-9	55,27,84,29	1-Penten-3-one		15.6	ND	ND	ND	6.61	ND	ND
1126	625-33-2	69,41,43,84	3-Penten-2-one		40.8^a^	ND	0.747^b^	ND	3.76^b^	ND	ND
1181	110-43-0	43,58,27,71	2-Heptanone	^P^	34.2^a,b^	0.492^b,c^	9.14^b^	3.57^b,c^	6.32^b,c^	0.478^c^	60.4^a^
1214	763-93-9	83,55,43,29	3-Hexen-2-one		16.1^a^	ND	ND	ND	0.592^b^	ND	ND
1238	928-68-7	43,58,41,71	2-Heptanone, 6-methyl-	^P^	11.9^b^	ND	1.25^b^	7.14^b^	3.10^b^	3.60^b^	48.2^a^
1285	111-13-7	43,58,41,71	2-Octanone	^P^	10.2	ND	ND	ND	3.15	0.753	9.09
1318	10408-15-8	43,58,41,68	1-Hepten-6-one, 2-methyl-		ND	ND	ND	2.82^b^	ND	ND	11.8^a^
1333	35194-31-1	68,43,67,58	6-Octen-2-one		67.8^a^	ND	ND	ND	1.79^b^	23.0^a^	ND
1339	110-93-0	43,41,69,55	5-Hepten-2-one, 6-methyl-		21.0^a^	15.7^a^	3.92^b^	3.41^b^	11.6^a,b^	12.0^a,b^	48.5^a^
1412	1669-44-9	55,43,111,41	3-Octen-2-one	^P^	13.2	ND	ND	ND	7.07	ND	ND
1525	38284-27-4	95,43,81,39	3,5-Octadien-2-one (*Z,Z*)-		205^a^	3.93^b^	2.36^b^	25.2^b^	29.5^b^	ND	ND
1536	7036-98-8	111,43,55,41	5-Nonen-4-one, 6-methyl-		ND	ND	ND	ND	ND	0.311	10.8
1579	30086-02-3	95,43,81,39	3,5-Octadien-2-one (*E,E*)-		134^a^	2.84^b^	6.08^b^	32.9^a,b^	10.4^b^	ND	ND
1604	77411-76-8	107,91,79	3,5-Heptadien-2-ona, 6-methyl-		ND	ND	ND	ND	ND	ND	15.3
**Cyclic ketones**					30.6^b^	3.41^c^	20.4^b^	64.5^b^	10.4^b,c^	3.07^c^	231^a^
1322	2408-37-9	82,56,69,55	Cyclohexanone, 2,2,6-trimethyl-		11.2^a^	1.05^b^	3.06^b^	18.9^a^	4.14^b^	2.48^b^	95.4^a^
1367	4694-12-6	83,56,69,41	Cyclopentanone, 2,4,4-trimethyl-		ND	0.481	ND	2.02	ND	ND	ND
1408	78-59-1	82,138,54,39	Isophorone^3^		ND	0.310^b^	0.742^b^	5.05^b^	0.656^b^	0.391^b^	33.2^a^
1708	1125-21-9	68,96,152,39	4-Oxoisophorone^4^		2.36^b,c^	0.215^c^	3.76^b,c^	22.2^a^	1.73^c^	0.202^c^	13.6^a^
1770	3859-41-4	28,42,98,56	1,3-Cyclopentanedione, 2-methyl-		12.3^a^	ND	7.13^a^	8.07^a^	0.34^b^	ND	ND
1879	6901-97-9	121,93,136	α-Ionone^5^	^P^	ND	0.0023^b,c^	0.118^c^	0.0633^c^	1.31^a^	ND	ND
1949	17190-74-8	166,43,109	Cinerolone^6^		ND	0.0208^b^	0.230^a^	0.547^a^	0.0606^b^	ND	0.317^a^
1973	14901-07-6	177,43,91	β-Ionone^7^	^P^	4.74^b^	1.33^b^	4.46^b^	7.72^b^	1.60^b^	ND	88.6^a^
**Nitrogen containing compounds**					172^a^	4.98^b^	26.9^a,b^	64.4^a^	5.84^b^	ND	318^a^
1267	109-08-0	94,67,40	Pyrazine, methyl-		13.2^a^	0.188^c^	3.07^b^	23.1^a^	ND	ND	15.8^a^
1281	100-71-0	106,107,79	Pyridine, 2-ethyl-		17.7^a^	0.193^c^	2.29^b,c^	3.15^b,c^	1.51^b,c^	ND	ND
1324	123-32-0	42,108,39,40	Pyrazine, 2,5-dimethyl-		ND	0.882^b^	3.30^b^	3.75^b^	ND	ND	238^a^
1331	118639	108,42,40,39	Pyrazine, 2,6-dimethyl-	^P^	132^a^	3.24^b^	4.93^b^	32.2^a^	ND	ND	56.7^a^
1387	13360-64-0	121,122,39	Pyrazine, 2-ethyl-5-methyl-		2.72	0.355	2.38	ND	ND	ND	3.52
1463	13925-07-0	135,136,42	Pyrazine, 2-ethyl-3,5-dimethyl-		ND	ND	0.903	ND	ND	ND	ND
1798	1453-58-3	82,81,54,27	1H-Pyrazole, 3-methyl-		5.47	ND	ND	ND	4.08	ND	ND
1917	541-46-8	59,44,41,43	Butanamide, 3-methyl		ND	ND	4.33^a^	0.0332^b^	ND	ND	ND
2289	21494-57-5	137,66	1H-Pyrrole-2,5-dione, 3-ethyl-4-methyl-		1.29	0.127	5.74	2.18	0.255	ND	4.46
**Sulfur containing compounds**					1400^a^	205^c^	928^b,c^	0.093^d^	ND	ND	0.119^d^
700	75-18-3	62,47,45,46	Methyl sulfide	^P^	767^a^	179^b^	2.18^c^	ND	ND	ND	ND
1592	67-68-5	63,78,45,61	Dimethyl sulfoxide		613^a^	25.3^b^	52.8^b^	ND	ND	ND	ND
1929	67-71-0	79,15,94,81	Dimethyl sulfone	^P^	21.0^a^	0.215^b^	16.1^a^	0.093^b^	ND	ND	0.119^b^
**Esters**					16.4^a^	19.5^a^	0.988^c^	0.766^c^	3.93^b,c^	19.2^a^	4.28^b^
824	79-20-9	43,74,42,59	Methyl acetate	^P^	ND	17.2	ND	ND	ND	18.8	ND
884	141-78-6	43,61,45,29	Ethyl acetate	^P^	ND	ND	ND	ND	3.44	ND	ND
1105	42125-10-0	43,68,67,86	2-Pentenyl acetate		8.64	1.61	ND	ND	ND	ND	ND
1358	16409-45-3	95,43,1,138	Menthyl acetate^8^		6.24	ND	0.572	ND	ND	ND	ND
2397	84-66-2	149,77,150	Diethyl phthalate^9^		1.43	0.684	0.416	0.766	0.485	0.368	4.28
**Furans**					292^a^	19.2^a,b^	24.2^a,b^	34.1^a,b^	67.4^a^	15.8^b^	299^a^
863	534-22-5	82,53,81,39	Furan, 2-methyl-	^p^	25.1^a^	2.51^b^	0.417^c^	1.31^b,c^	2.49^b^	8.97^b^	8.58^b^
950	3208-16-0	81,53,96,39	Furan, 2-ethyl-	^P^	180^a^	9.78^b,c^	2.50^c^	8.03^b,c^	25.4^b^	1.70^c^	ND
1030	4229-91-8	81,83,110,27	Furan, 2-propyl-		8.37	1.20	ND	ND	0.840	1.57	ND
1229	3777-69-3	81,82,138,53	Furan, 2-Pentyl-		78.6^a^	5.76^b^	17.9^b^	18.7^b^	38.7^a,b^	3.57^b^	271^a^
1522	81250-44-4	137,43,152	5-Isopropyl-3,3-dimethyl-2-methylene-2,3-dihydrofuran		ND	ND	3.34^b^	6.04^a,b^	ND	ND	19.4^a^
**Miscellaneous**					146^b^	35.8^c^	93.3^b,c^	145^b^	59.8^c^	142^b^	422^a^
850	541-05-9	207,208,96	Cyclotrisiloxane, hexamethyl-	^P^	65.0^a^	8.95^a,b^	4.31^b^	38.5^a^	22.4^a^	1.23^b^	18.3^a^
912	13417-43-1	69,921,104	2-Butene, 1-chloro-2-methyl-		32.8	3.33	9.41	18.1	ND	ND	6.37
926	75-09-2	49,84,86,51	Methylene chloride		ND	ND	ND	ND	ND	2.57	ND
963	142-96-1	57,41,29,56	Butyl ether		ND	ND	ND	ND	0.953^b^	51.8^a^	ND
1017	67-66-3	83,85,47,48	Trichloromethane	^P^	43.6^b^	22.8^b^	30.6^b^	84.1^a,b^	34.8^b^	86.2^a,b^	341^a^
1727	503-74-2	60,43,41,45	Butanoic acid, 3-methyl-		ND	ND	34.2	ND	ND	ND	ND
2031	23267-57-4	123,43,41	β-Ionone, 5,6-epoxy-^10^	^P^	4.13^b^	0.449^b^	14.8^a,b^	4.24^b^	0.773^c^	ND	56.5^a^
**Total volatile compounds**					4704^a^	546^c^	592^c^	1920^b^	753^b,c^	760^b,c^	6697^a^

* LRI* linear retention index, *ND* not detected.

*Ions selected for quantification; ^P^positive identification with pure standards; ^a,b,c,d^Means with different superscripts indicate statistically significant (P ≤ 0.05) differences among strains. ^1^1-cyclohexene-1-carboxaldehyde, 2,6,6-trimethyl-; ^2^1,3-cyclohexadiene-1-carboxaldehyde, 2,6,6-trimethyl-; ^3^2-cyclohexen-1-one, 3,5,5-trimethyl-; ^4^2-cyclohexene-1,4-dione, 2,6,6-trimethyl-; ^5^3-buten-2-one, 4-(2,6,6-trimethyl-2-cyclohexen-1-yl)-; ^6^2-cyclopenten-1-one, 2-(2-butenyl)-4-hydroxy-3-methyl-, (*Z*)-; ^7^3-buten-2-one, 4-(2,6,6-trimethyl-1-cyclohexen-1-yl)-; ^8^cyclohexanol, 5-methyl-2-(1-methylethyl)-, acetate; ^9^1,2-benzenedicarboxylic acid, diethyl ester; ^10^3-buten-2-one, 4-(2,2,6-trimethyl-7-oxabicyclo[4.1.0]hept-1-yl).

**Table 2:** Percentage of relative abundance (%) and number of volatile compounds (N) corresponding to chemical families found in selected strains of *Isochrysis galbana (IG)*, *Nannochloropsis gaditana (NG), Tetraselmis* sp. *(TS), Scenedesmus almeriensis (SA)*, *Chlorella vulgaris (CV) Synechococcus* sp. (*SY*) and *Arthrospira platensis* (*AP*) classified by domain (eukaryote-microalgae and prokaryote-cyanobacteria) and environment (marine and freshwater).

Chemical family	Eukaryote										Prokaryote			
	Marine						Freshwater				Marine		Freshwater	
	*IG*		*NG*		*TS*		*SA*		*CV*		*SY*		*AP*	
	%	N	%	N	%	N	%	N	%	N	%	N	%	N
Acyclic aldehydes	18.2^a,b^	17	2.79^c^	7	10.1^b,c^	13	1.88^c^	7	21.7^a^	16	6.40^c^	6	4.82^c^	3
Cyclic aldehydes	0.849^b,c^	2	0.199^c^	1	3.70^a^	3	1.30^b^	3	0.905^b^	3	0.111^c^	1	0.669^b,c^	3
Aromatic hydrocarbons	2.78^b^	4	8.77^a,b^	3	14.0^a,b^	3	12.0^a,b^	3	18.0^b^	4	24.4^a^	3	10.1^b^	2
Branched hydrocarbons	2.54^b^	2	2.67^b^	2	2.11^b^	2	46.2^a^	40	2.09^b^	4	8.92^b^	7	37.3^a^	39
Alicyclic hydrocarbons	0.854^b^	2	0.212^b^	1	0.968^b^	3	0.600^b^	4	0.553^b^	2	2.84^a^	4	0.423^b^	3
Linear hydrocarbons	3.00^b^	4	6.21^a,b^	4	4.99^b^	4	7.37^a,b^	6	5.68^a,b^	4	22.4^a^	6	12.9^a,b^	9
Acyclic alcohols	11.3^a,b^	9	18.6^a^	8	11.2^a,b^	8	7.20^c^	6	15.6^a,b^	7	0.652^e^	3	5.55^d^	6
Cyclic alcohols	1.06^c^	2	1.30^c^	2	7.48^a^	2	3.24^b^	2	2.04^b,c^	2	0.272^d^	1	3.00^b^	2
Acyclic ketones	15.9^a^	15	6.69^c^	8	5.51^c^	10	4.05^c^	7	13.9^a^	15	10.4^b,c^	8	6.26^c^	9
Cyclic ketones	0.647^c^	4	0.625^c^	7	3.44^a^	8	3.36^a^	8	1.39^b^	8	0.404^b,c^	3	3.45^a^	5
Nitrogen containing compounds	3.64^b^	6	0.913^c^	6	4.54^a,b^	8	3.36^a^	6	0.781^c^	3	ND		4.75^a^	5
Sulfur containing compounds	29.6^a^	3	37.5^a^	3	12.0^b^	3	0.005^c^	1	ND		ND		0.002^c^	1
Esters	0.345^b^	3	3.57^a^	3	0.166^b^	2	0.040^b^	1	0.524^b^	2	2.53^a^	2	0.064^b^	1
Furans	6.17^b^	4	3.53^b,c^	4	4.07^b,c^	4	1.77^c^	4	9.00^a^	4	2.08^b,c^	4	4.46^b,c^	3
Miscellaneous	3.07^b^	4	6.51^b^	4	15.7^a^	5	7.55^b^	4	7.86^b^	4	18.7^a^	4	6.30^b^	4
Total VOCs		81		63		78		102		78		52		95

* VOCs* volatile organic compounds, *ND* not detected.

^a,b,c,d,e^Means with different superscripts indicate statistically significant (*P* ≤ 0.05) differences among strains.

Overall, 150 volatile compounds were detected in the seven strains studied (Table 1). The number of detected VOCs described in previous bibliography is quite variable. Van Durme et al.^9^ detected 57 compounds in five microalgal strains (*Botryococcus braunii, Rhodomonas, Tetraselmis *sp.,* Nannochloropsis oculata* and *Chlorella vulgaris*), Zhou et al.^10^ described 246 compounds in six microalgal strains during three growth phases (*Thalassiosira weissflogii, Nitzschia closterium, Chaetoceros calcitrans, Platymonas helgolandica, Nannochloropsis *sp. and *Dicrateria inornata*), and de Jesus et al.^11^ detected only 29 compounds in *AP*. This wide variation in the number of identified compounds may be due to the low number of the studies reported to date and differences in the methodologies used for volatile extraction and analysis.

Total number of VOCs varied significantly among strains being *AP* and *Scenedesmus almeriensis (SA)* the richest strains with 95 and 102 VOCs respectively. *Isochrysis galbana (IG)* (81), *CV* (78), *Tetraselmis *sp. *(TS)* (78), *Nannochloropsis gaditana (NG)* (63), and *Synechococcus* sp. (*SY)* (52) presented lower VOCs diversity (Table 2). These results highlighted the high variability in both individual volatile compounds and chemical families among different microalgae. Results were in line with those described by Zhou et al.^10^ who observed variations ranging between 46 and 84 compounds depending on microalgal strain and growth phase. In terms of relative abundance (RA), *IG* and *AP* were the richest species (*P* ≤ 0.05) for total VOC abundance (Table 1). To the best of the authors’ knowledge, this is the first report describing the volatile profile of *SA*, *SY*, and *IG*. As a whole, the main chemical families in terms of number of compounds for all strains analysed were branched hydrocarbons (40), aldehydes (18) and ketones (17) (Table 2). These three chemical families have been previously reported as major chemical classes in microalgae^9,10^.

### Aldehydes

*IG* and *CV* were the richest species in both number of aldehydes and percentage of RA (17 compounds and 18.2%, and 16 compounds 21.7%, respectively; Table 2). The number of acyclic aldehydes was very variable between the different strains (between 3 and 18 compounds). In turn, only 3 cyclic aldehydes were detected, being their percentage of relative abundance only relevant in *TS* (3.7%). Only shortchain aldehydes (between 6 and 9 carbons) were found in the selected strains probably due to the low extraction temperature used (30 ºC)^12^. In terms of RA, the microalgal strain with the highest content (*P* ≤ 0.05) of acyclic aldehydes was *IG* followed by *AP* and *CV* (Table 1). Again, *AP* showed a fairly extraordinary situation with very few aldehydes detected and a very high content of propanal, 2-methyl-, 2-pentenal, 2-methyl-, hexanal, and 2-butenal, 2-methyl- were the most abundant aldehydes in *IG,* while the RA of hexanal was five times higher than that of any other aldehyde in *CV* as reported in previous studies^9^_._ Particularly noteworthy was the high RA of 2,4-heptadienal, (*E,E*)- in *SY*. Among cyclic aldehydes, benzaldehyde was the major compound in all species with the exception of *AP* that was very rich in β-cyclocitral (Table 1).

Short-chain aldehydes (alkanals, alkenals, and alkadienals) are formed from fatty acid autoxidation (mainly long-chain fatty acids with more than 18 carbon atoms)^13^. On the other hand, benzaldehyde and butanal, 3-methyl- have been related with amino acid degradation^9,14^, and β-cyclocitral together with β-ionone are derived from enzymatic degradation of β-carotene^15^. Aldehydes can contribute to microalgal and cyanobacterial odour because most of them show low odour threshold (OT) values (Table 3). Overall, saturated aldehydes have been related with green-like and hay-like odour notes whereas unsaturated aldehydes can impart fatty and oily odours^9^. Butanal, 3-methyl-, hexanal, heptanal, 2-octenal, (*E*)-, and β-cyclocitral were the aldehydes with higher odour impact values in most of the strains while propanal, 2-methyl- was the most important odorant exclusively in *AP*. β-Cyclocitral has been identified as a powerful odorant and one of the most abundant VOCs in cyanobacteria^16^. It was found in all species except for *NG* and *SY*, as previously reported^9^.

**Table 3:** Estimated mean odour impact ratio (OIR) values for volatile compounds detected in *Isochrysis galbana (IG)*, *Nannochloropsis gaditana (NG), Tetraselmis* sp. *(TS), Scenedesmus almeriensis (SA)*, *Chlorella vulgaris (CV) Synechococcus* sp (*SY*) and *Arthrospira platensis* (*AP*).

Compounds	OT (mg/kg)	Ref.	OIR						
			*IG*	*NG*	*TS*	*SG*	*CV*	*SY*	*AP*
Pyrazine, 2-ethyl-3,5-dimethyl-	0.000	^36^	–	0	22,575	–	–	–	–
β-Ionone	0.0001	^37^	35,111	9852	33,037	57,185	11,852	–	656,296
Butanal, 3-methyl-	0.001	^27^	19,727	–	10,727	8455	8345	–	–
Methyl sulfide	0.001	^38^	697,272	162,727	1982	–	–	–	–
1-Octen-3-ol	0.002	^27^	57,400	4353	18,533	5693	22,800	–	43,867
Propanal, 2-methyl-	0.002	^27^	–	–	1527	–	–	–	205,333
Butanal	0.002	^39^	3435	–	418	–	945	–	–
Furan, 2-ethyl-	0.002	^27^	78,261	4252	1087	3491	11,043	739	–
Heptanal	0.003	^27^	8607	400	654	–	1111	320	2386
2-Octenal, (*E*)-	0.003	^27^	3020	200	103	–	1297	–	–
β-Cyclocitral	0.003	^40^	1083	–	1773	1527	343	–	7000
4-Heptenal, (*Z*)-	0.004	^27^	4595	–	–	–	307	–	–
α-Ionone	0.004	^37^	–	1	31	17	347	–	–
Hexanal	0.005	^27^	28,400	1964	5780	–	14,540	774	–
Ethyl acetate	0.005	^27^	–	–	–	–	688	–	–
1-Hexanol	0.006	^27^	1273	221	480	–	380	–	26,607
Furan, 2-Pentyl-	0.006	^27^	13,322	976	3034	3169	6559	605	45,914
2-Heptanone, 6-methyl-	0.008	^41^	1469	–	154	881	383	444	5951
Propanal	0.015	^27^	3596	67	99	301	828	–	–
2,4-Heptadienal, (*E,E*)-	0.015	^33^	2318	–	–	156	870	2623	–
Pyrazine, 2-ethyl-5-methyl-	0.016	^42^	170	22	149	–	–	–	220
1-Penten-3-one	0.023	^43^	678	–	–	–	287	–	–
2,3-Pentanedione	0.029	^44^	1007	27	34	–	75	–	–
2-Octanone	0.050	^27^	203	–	–	–	63	15	181
Benzene, ethenyl-	0.065	^45^	655	168	523	–	285	540	–
5-Hepten-2-one, 6-methyl-	0.068	^27^	309	231	58	50	171	176	713
2-Hexenal, (*E*)-	0.082	^46^	96	–	8	–	20	–	–
2-Penten-1-ol, (*E*)-	0.089	^27^	274	25	–	33	–	–	–
2,4-Heptadienal, (*E,Z*)-	0.095	^27^	40	–	–	–	35	–	–
3,5-Octadien-2-one (*E,E*)-	0.100	^47^	1340	28	61	329	104	–	–
Cyclohexanone, 2,2,6-trimethyl-	0.100	^48^	112	11	31	189	41	25	954
2-Heptanone	0.140	^49^	244	4	65	26	45	3	431
1-Pentanol	0.150	^27^	104	1	26	–	15	–	204
Furan, 2-methyl-	0.200	^35^	126	13	2	7	12	45	43
Isophorone	0.200	^32^	–	2	4	25	3	2	166
2-Pentenal, 2-methyl-	0.290	^32^	876	4	5	16	34	–	–
2-Hexen-1-ol, (*Z*)-	0.359	^27^	48	19	17	13	11	2	331
Pyrazine-2,6-dimethyl-	0.400	^42^	330	8	12	81	–	–	142
2-Butanol, 3-methyl-	0.410	^27^	–	–	4	–	7	–	–
1-Butanol	0.459	^27^	–	–	–	–	–	6	–
2-Butenal, 2-methyl-	0.459	^27^	296	2	13	24	14	–	–
Butanoic acid, 3-methyl-	0.490	^38^	–	–	70	–	–	–	–
2-Penten-1-ol, (*Z*)-	0.720	^50^	79	22	5	16	9	–	–
Benzaldehyde	0.751	^27^	49	1	17	17	7	1	14
2-Pentenal, (*E*)-	0.980	^46^	13	0	0	–	5	1	8
3-Penten-2-one	1.20	^50^	34	–	1	–	3	–	–
1-Penten-3-ol	1.46	^27^	215	47	14	75	44	1	5
Methyl acetate	1.50	^41^	–	11	–	–	–	13	–
Pyrazine, 2,5-dimethyl-	1.75	^49^	–	1	2	2	–	–	136
Benzene, ethyl-	2.21	^27^	3	1	1	0	1	2	–
Pentanal	2.50	^27^	9	–	2	1	6	1	–
Octane	10.0	^51^	5	–	0	1	0	1	2
Nonane	10.0	^51^	–	–	–	1	–	–	4
Decane	10.0	^51^	–	–	0	6	0	–	22
Undecane	10.0	^51^	–	–	–	1	–	–	2
Pyrazine, methyl-	30.0	^42^	0	0	0	1	–	–	1
2-Butanone	35.4	^27^	1	0	0	–	0	0	–
2-Propanone	45.0	^27^	1	0	0	–	0	1	5
Heptane	50.0	^51^	1	1	1	1	1	1	3
2-Pentanone	75.0	^27^	1	0	0	0	0	–	0
Safranal	n/a		–	–	–	–	–	–	–
Menthyl acetate	n/a		–	–	–	–	–	–	–
3-Octen-2-one	n/a		–	–	–	–	–	–	–
3,5-Octadien-2-one	n/a		–	–	–	–	–	–	–
4-Oxoisophorone	n/a		–	–	–	–	–	–	–
Pyridine, 2-ethyl-	n/a		–	–	–	–	–	–	–
Dimethyl sulfoxide	n/a		–	–	–	–	–	–	–
Dimethyl sulfone	n/a		–	–	–	–	–	–	–
β-Ionone, 5,6-epoxy-	n/a		–	–	–	–	–	–	–

* OT* odour thresholds in water, *Ref.* bibliographic reference, *n/a* not available bibliographic threshold.

### Hydrocarbons

**Figure 1:**
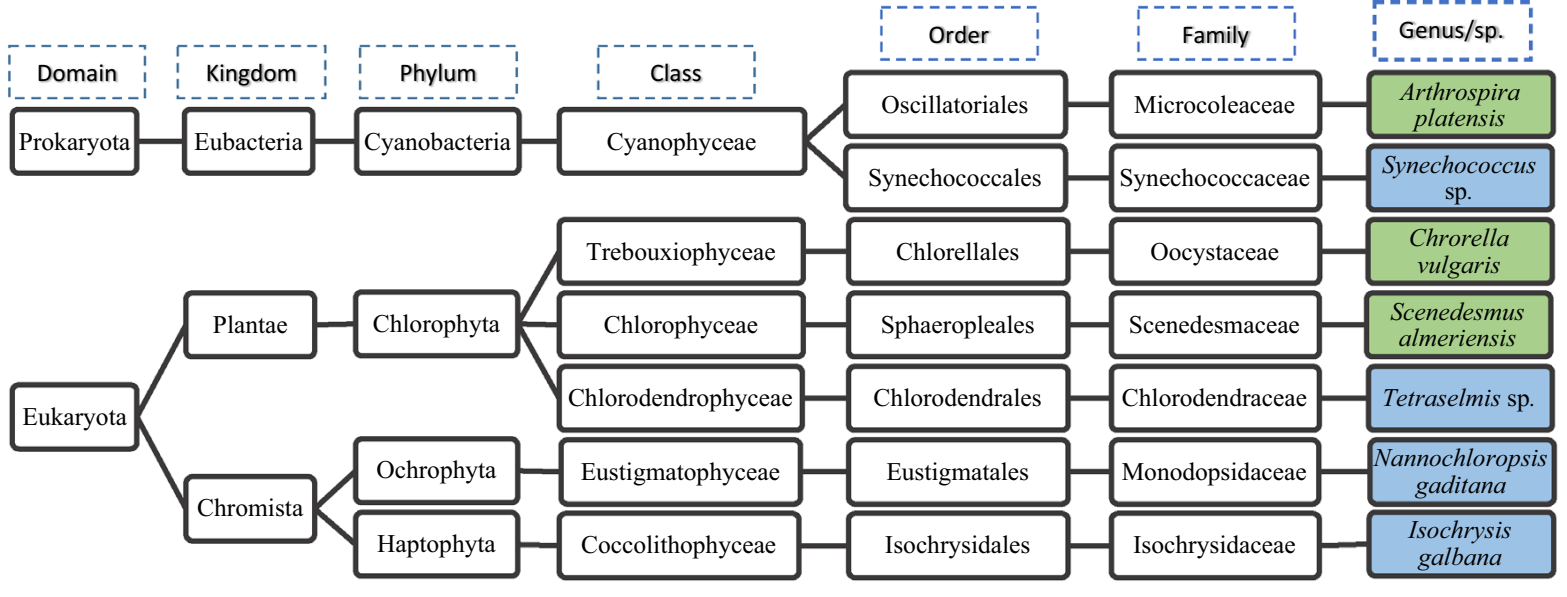
Taxonomic classification of studied microalgal and cyanobacterial strains. Specie/Gender in green are related with freshwater while those in blue are representative of marine environments.

A total of 12 linear, 4 aromatic, 40 branched, and 5 alicyclic hydrocarbons were identified in the studied microalgal and cyanobacterial strains. These hydrocarbons showed a moderated variation in both the number of individual compounds and percentage of relative abundance among strains. The most remarkable variability was in branched hydrocarbons which only occurred in two freshwater species (*SA* and *AP*). These species presented 40 and 39 branched compounds, respectively, whereas the maximum number detected in the other species ranged between 2 and 7 individual compounds. Similarly, branched hydrocarbons represented 2–9% of total VOCs relative abundance in most strains, although the abundance of these compounds in *SA* and *AP* was much higher (*P* ≤ 0.05) (46.2 and 37.3% of total VOCs, respectively; Table 2). It is important to highlight that, in the present work, some branched hydrocarbons were only identified to chemical family level due to the similarity among mass spectra and linear retention index (LRI) between branched compounds, the limited bibliographic information on LRI values, and the scarce availability of commercial high purity compounds. This noteworthy VOCs present in *AP* was overlooked in the scarce bibliography available, despite the presence of branched hydrocarbons have been highlighted in superior algae^17^ such as *Capsosiphon fluvescens*^18^ and *Undaria pinnatifid* ^19^ or even in other microalgae such as *Nostoc* sp.^20^. On the other hand, it is also remarkable the similarity of the VOCs profile of *AP* and *SA*, even though *SA* is a eukaryotic microalga and *AP* a prokaryotic cyanobacterium (Fig. 1). The individual chromatographic VOCs profile of each strain is provided as Supplementary Table S1. Among individual compounds, a larger proportion of large linear hydrocarbons (more than 13 carbon atoms) were found in prokaryotic cyanobacteria (Table 1) as reported previously^16^. In this respect, some of these linear hydrocarbons could have contaminated the samples during the production and freeze-drying process of strains in the laboratory, or during storage of the samples in plastic containers.

The origin of branched hydrocarbons has been previously related with the oxidation of branched-chain fatty acids in other food matrices^21^. However, it should be noted that in some microalgae species such as *AP*, very low branched-chain fatty acid contents have been reported^22^. Therefore, it is likely that these compounds are coming from a secondary route, but further research is needed to understand the origin of these compounds. In general, acyclic hydrocarbons have been described to have no significant flavour contribution in food matrices^13^ and it was confirmed by the odour impact ratio (OIR) values estimated in the selected strains (Table 3). Moreover, the contribution of branched hydrocarbons to flavour is still quite uncertain as there are no OT values available for these compounds in specialised databases or the scientific literature.

Aromatic hydrocarbons can be assumed to be generated mainly from the degradation of aromatic amino acids^23^. They are usually known as important aromatic compounds. However, in the present work, the only one that seemed to impact in a moderate way (OIR values from 168 to 655) was benzene, ethenyl- (Table 3).

### Ketones

Acyclic (18) and cyclic (8) ketones were deemed a very representative chemical class in some of the studied strains. In this regard, *IG* and *CV* were the microalgae with the highest number of individual acyclic ketones (15 each) and also the highest percentage of RA (*P* ≤ 0.05) (15.9 and 13.9%, respectively). In turn, cyclic ketones were more representative of the VOCs profile in *TS*, *SA*, and *AP* in both number of compounds and percentage of RA (Table 2). In macroalgae or seaweed, acyclic ketones are usually related with less evolved brown seaweeds, while cyclic ketones are related with more evolved species^19^. In the current study, cyanobacterial strains showed a lower number of cyclic ketones than most microalgae (Table 2). The relative abundance of acyclic ketones was significantly higher in *IG* when compared to the other strains (Table 1), mainly due to the extremely high abundance of 3,5-octadien-2-one (*E,E*)- and (*Z,Z*)- in the former. Both configuration isomers were also present as major ketones in *SA* and *CV* and they were detected exclusively in eukaryote microalgae whereas among prokaryote strains, the major ketone was 2-propanone (Table 1). Van Durme et al.^9^ reported 1-penten-3-one, 3-pentanone and 2-butanone, 3-methyl- as major ketones in microalgae species. In addition, acyclic ketones were very similar between marine and freshwater strains in both, relative abundance and number of individual compounds.

Branched cyclohexanones and cyclopentanones were major compounds among cyclic ketones in all the studied strains. Cyclohexanone, 2,2,6-trimethyl- has been previously reported as a characteristic ketone in cyanobacteria^23^. Isophorone was present in both cyanobacteria and 4-oxoisophorone was a major ketone in *CV* and *SA*. Furthermore, 1,3-cyclopentanedione, 2-methyl- showed significantly (*P* ≤ 0.05) higher RA in *IG*, *TS*, and *SA* than in the other strains. In line with the high relative abundance (*P* ≤ 0.05) of β-ionone found in *AP*, cyanobacteria were previously featured as a rich source of carotenoids^23^. However, the results of the present work indicated that the presence of nor-carotenoids was strain-dependent, since β-ionone was not detected in the *SY*.

The origin of acyclic ketones is variable, while linear ketones are derived from lipid oxidation, some methyl ketones may result from β-oxidation of the fatty acids and subsequent decarboxylation and others are mainly products of oxidative cleavage of carotenoids as above-mentioned for β-cyclocitral^25^. In general, acyclic ketones are related with desirable odours in food. Saturated ketones are related with sweet, floral, and fruity odour notes, while unsaturated ketones are responsible for green odour notes^10^. Moreover, Van Durme et al.^9^ related the seafood-like odour in microalgae to their high content in diketones such as 2,3-pentanedione and 3,5-octadien-2-one. In the present work, these compounds were found in considerable amounts in microalgae, while the only diketone found in cyanobacteria was 3,5-heptadien-2-ona, 6-methyl- (exclusively in *AP*). Prokaryotic chlorophytes such as *AP* have been previously related with high amounts of methyl-ketones such as 2-propanone, 2-pentanone, 2-peptanone and 2-octanone which contribute to green odour notes^24^. Attending to OIR values (Table 3), acyclic ketones generated a great odour impact mainly in *IG* and *AP*. 2,3-Pentanedione, 2-heptanone, 6-methyl-, and 3,5-octadien-2-one (*E,E*)- presented the highest OIR values in *IG* while 2-heptanone, 6-methyl- was also deemed important in *AP*. The latter compound has been included in the camphoreous odour family (Supplementary Table S1). Among cyclic ketones, β-ionone was the major odorant in freshwater strains, and one of the most abundant odorants in marine strains (Table 3). Previous studies reported β-ionone as a potent odorant in some microalgae such as *Scenedesmus* sp. and *CV* and also in macroalgae^25,26^. In addition, this compound has been related to the characteristic odour of microalgae^10,25^ together with β-ionone, 5,6-epoxy-.

### Alcohols

Alcohols in microalgae are mainly formed as secondary decomposition of hydroperoxides of fatty acids, with the exception of branched alcohols that may also derive from carbohydrates via glycolysis or from amino acids through the Ehrlich pathway^27^. In the present study, marine eukaryotes *IG*, *NG*, and *TS* were the strains presenting higher diversity of acyclic alcohols with 9, 8, and 8 individual compounds detected respectively. The number of cyclic alcohols found in these species was lower and similar between them (Table 2). Similarly, the percentage of relative abundance of acyclic alcohols was, in general, significantly higher in marine (11.2–18.6%) than in freshwater strains (7.2–15.6%), except for *CV* that presented a high proportion of acyclic alcohols (15.6%). Overall, the abundance in cyanobacteria was significantly smaller (0.65–5.55%) when compared with microalgal strains. The diversity of cyclic alcohols was similar among the studied strains and *TS* presented the highest proportion (*P* ≤ 0.05) of cyclic alcohols (7.48%) (Table 2). Regarding individual compounds, 1-penten-3-ol, 2-penten-1-ol, (*Z*)-, and 1-octen-3-ol were major compounds in all eukaryote strains while very different alcohol composition was observed between prokaryotes. Briefly, *SY* showed negligible amounts of both acyclic and cyclic alcohols in comparison to the other strains (*P* ≤ 0.05) whereas major alcohols in *AP* were 1-hexanol, 2-hexen-1-ol, (*Z*)- and cyclohexanol, 2,4-dimethyl-. It is remarkable the absence of geosmin and isoborneol, 2-methyl- in freshwater microalgae and specially in cyanobacterial strains since these two compounds have been previously reported as odorant compounds related to earthy-muddy odour notes in cyanobacterial^24^. Commercial high purity standards of both compounds were analysed in order to ensure their absence in cyanobacteria strains. These results were comparable to those of previous reports in which geosmin and isoborneol, 2-methyl- were no detected in *AP*^16^ nor in other freshwater species^9^. Results reported herein were in line with previous research on VOCs content in microalgae, where *TS* presented lower content of alcohols when compared to *CV* and *NG* (Table 1)^9^. In contrast, Van Durme et al.^9^ found low amounts of 1-octen-3-ol while in the present study this acyclic alcohol was major in most of the studied strains as in dehydrated edible seaweed^19^. Although alcohols have relatively high OT values, some unsaturated alcohols may exert an important impact in odour^20^. In the present work, 1-octen-3-ol (earthy, green, oily, fungal, grassy, and fatty) was deemed as one of the compounds with higher OIR in most species (Table 3).

### Nitrogen and sulfur containing compounds

Nitrogen- and sulfur-containing compounds were also detected in the headspace of the selected strains. Sulfur compounds were deemed as major compounds (*P* ≤ 0.05) (in terms of relative abundance; Table 1) in marine microalgae, particularly in *IG* and *NG* where they represented 29.6 and 37.5% of total VOCs, respectively (Table 2). In turn, sulfur compounds were not detected, or detected in negligible abundance, in cyanobacteria and freshwater strains. The most abundant sulfur compound in microalgae was methyl sulfide, although the abundance of dimethyl sulfoxide was also important in some species such as *IG* (Table 1). Nitrogen compounds abundance was lower than that of other chemical families and only in *IG, TS*, *SA* and *AP* reached percentages between 3 and 5% of total VOCs abundance (Table 2).

The formation of sulfur compounds has been previously related with catabolism of free, peptide and protein sulfur-containing amino acids^28^, while in marine algae the presence of dimethyl sulfide was related to the degradation of sulfonio propionate, dimethyl-^18^. On the other hand, nitrogen compounds are commonly described as derived from Maillard reactions due to high temperatures applied to food or biological matrices^27,28^, and pyrazines and pyridines have been reported as abundant in dried seaweeds^29^. In the present work, the microalgal strains were dehydrated by freeze-drying and the temperature used for volatile extraction was low (30 ºC). Therefore, it is unlikely that those procedures could originate thermal-derived nitrogen compounds. Probably, the pyrazines found in the headspace of microalgae and cyanobacteria could be originated from trimethylamine N-oxide degradation during storage^15^.

Both nitrogen- and sulfur-containing compounds have been related to fish odours^10,13^. Sulfur compounds were associated with the characteristic aroma of marine crustaceous^13^ while alkylpyrazines generally deliver roasty odour notes^28^ and nutty odour such as the case of pyrazine, methyl- (Supplementary Table S1). In fish, this compound has been related with fishy and ammonia odours. Nitrogen compounds seem to contribute little to the final odour due to their low OIR values (lower than 100) in most species (Table 3). On the contrary, the low OT values for methyl sulfide together with its high abundance in marine microalgae strains make methyl sulfide the most important odorant in these species (Table 3). In this regard, previous studies have reported that sulfur compounds were responsible for characteristic odours of marine microalgae such as cooked shrimp/cooked seafood and marine and fishy odours^9^.

### Esters, furans and other compounds

The RA of the esters in the strains was small and each individual compound seemed to be characteristic of each specie. Ethyl and methyl acetate were previously reported in microalgae biomass^10^. Diethyl phthalate was found in all the studied strains, being phthalate products regarded as toxic pollutants^18^.

Furans were found as constant compound present in microalgae VOC composition. The number of furan compounds was very similar in both marine and freshwater strains although a significantly (*P* ≤ 0.05) higher percentage of RA was observed in *CV* (9.00%) in comparison with the rest of the species (Table 2). The abundance of furan 2-methyl-, furan, 2-pentyl-, and furan, 2-ethyl- was particularly considerable in *IG, TS*, and *CV* (Table 1). Moreover, furans have been reported previously as microalgae VOCs^9,16^. Furans can be formed by Amadori pathways from the oxidation of fatty acids or by glucose pyrolysis^28^. In general, furans have been identified as off-flavours of fat and oils imparting a beany, grassy liquorice, and tobacco odour notes^27^.

## Materials and methods

### Microalgae and cyanobacteria strains selection and production

Selected strains included *Isochrysis galbana* (*IG—*REC 0002B), *Nannochloropsis gaditana* (*NG—*BEA 1202)*, Tetraselmis* sp. (*TS—*BEA 0098/2)*, Chlorella vulgaris* (*CV—*CCAP 1475/9), *Synechococcus* sp. (*SY—*PCC 7942), *Arthrospira platensis* (*AP—*BEA 0005B), and *Scenedesmus almeriensis* (*SA—*CCAP 276/24), which is a lutein overproducing strain isolated by the Chemical Engineering Department of the University of Almería (Spain). Selected strains were produced in controlled closed bubble column photobioreactors located inside a greenhouse at the pilot plant facilities of the University of Almería. Full taxonomic characteristics of the strains used in the present work are provided in Fig. 1.

Daily maximum, minimum, and average temperature inside the greenhouse were 27.0 ± 2.2, 11.7 ± 1.7 and 18.4 ± 1.7 °C, respectively. Average irradiance during the approximately 12 h of sunlight was 600.2 ± 72.2 µE/m^2^·s with peaks of 1500–1600 µE/m^2^·s at midday. The pH of all the strains except for *AP* was controlled by on-demand injection of carbon dioxide at 8.0. Culture media used for the production of *CV* and *SA* was the Arnon medium^30^ for *AP* the Arnon medium supplemented with sodium bicarbonate (16.8 g/L; pH 9.5 ± 0.2) and for *NG*, *TS, IG* and *SY* the Algal medium^31^. Once the biomass concentration reached approximately 1.5 g/L, the biomass was harvested and concentrated by centrifugation using a Sigma 3–18 KS centrifuge (Sigma Laborzentrifugen, Osterode am Harz, Germany) operating at 8000*g* for 10 min. The concentrated biomass with a concentration of approximately 20 g/L was immediately frozen at −80 ºC and freeze-dried using a Crydos-50 freeze-dryer (Telstar, Barcelona, Spain). The obtained dried powder was stored in a sealed plastic container at room temperature until further analysis.

### Solid-phase microextraction of volatile compounds

Detailed description of the chemicals and suppliers used in the present experiment are described in the Supplementary Data.

Freeze dried microalgal/cyanobacterial biomass was weighted (0.300 ± 0.001 g) in triplicate in 10 mL amber vials (Agilent Technologies, Madrid, Spain) and 10 μL internal standard (IS) were added (0.1 mg/mL of cyclohexanone in hexane solution). Vials were subsequently sealed with PTFE septa and a steel magnetic cap (18 mm PTFE/SIL, Agilent Technologies), vortexed for 15 s, and left in a chilled room (4 ± 1 ºC) for 24 h prior to analysis.

The solid-phase microextraction procedure was performed using a PAL RSI 85 autosampler (CTC Analytics, Zwingen, Switzerland). After 15 min of pre-equilibration time at the extraction temperature, volatile compounds were trapped onto a 1 cm long divinylbenzene/carboxen/polydimethylsiloxane fiber (57298-U, 50/30 µm, Supelco, Madrid, Spain) at 30 ºC for 30 min.

Volatile compounds trapped onto the fiber were desorbed in the front injection port of the GC equipment for 15 min at 240 °C in splitless mode (split valve was opened at 200 mL/min after 10 min of the injection) using the autosampler device. After thermal desorption, the fiber was directly cleaned in the back injection port for 30 min at 270 °C.

The working routine of the automatic sampler was to perform a blank (empty 10 mL amber vial) every three sample analyses. All the microalgal and cyanobacterial samples were analysed on the same day, and the samples were randomly located in the autosampler tray.

### Gas chromatography–mass spectrometry analysis

Volatile compounds were analysed using a 7820A gas chromatograph (Agilent Technologies) equipped with two split/splitless injectors and coupled to a 5975 series mass spectrometry detector (Agilent Technologies). The volatile compounds were separated in a Supelcowax-10 (Supelco) fused silica capillary column (60 m long, 0.25 mm i.d., 25 µm film thickness) as described in Moran et al.^12^.The mass spectrometer consisted in a single quadrupole operating in full scan mode (1.4 scans/s, m/z range 26–350) at 230 ºC with a total ion current of 70 eV.

Chromatographic data were analysed with MSD ChemStation Data Analysis (version 5.52, Agilent Technologies). The limit of detection (LOD) was calculated from the noise obtained in the analysis of ten blanks. LOD was set as twice the average noise for each chromatographic zone. Mean linear retention index (LRI) values were calculated using the average real retention time of three replicates of each compound and the retention time of the standard saturated alkanes certified reference material. LRI values showed a variation coefficient less than 0.15% for all individual volatile compounds.

Tentative identification of volatile compounds was performed by comparing their mass spectra (matching factor > 800) with those of the National Institute of Standards and Technology (NIST version 2.0, Gaithersburg, USA). Additionally, peak identifications were confirmed by comparison of experimental LRI values with those previously published for volatile compounds analysed under similar chromatographic conditions when available. Positive identification was performed by comparison of the experimental LRI and mass spectra with those of commercial standards. Chromatographic peak areas were measured using selective integration for the four more abundant *m/z* ions of each target compound according to NIST mass spectra.

Peak areas (> LOD) of individual volatile compounds detected in at least two of the three replicates were used to calculate mean abundances in each sample. The volatile compound content of the samples was expressed as relative abundance (RA, arbitrary area units) to the area of IS according to the following equation:

$$\text{RA} = \frac{\text{peak area}}{\text{IS area}} \, {\times} \, \frac{\text{0.3 g}}{\text{sample weight (g)}} \, {\times}\text{ 100}$$

Peak areas were multiplied by 10^–5^ for easier comprehension and three significant figures were used to express the RA of volatile compounds in the samples.

### Odour impact ratio of volatile compounds

The odour intensity of the different volatile compounds identified was estimated by means of the odour impact ratio (OIR). Briefly, available odour threshold (OT) values measured in water were collected from available databases^32–34^, and the OIR for the individual volatile compounds was calculated as follows:

$$\mathrm{OIR }= \frac{\mathrm{mean RA}}{\mathrm{OT }({\upmu {\rm g}}/\mathrm{kg})}$$

Additionally, odour notes for volatile compounds were described according to The Good Scents Company database^35^ and Giri et al.^27,28^ and are provided in Supplementary Table S1.

### Statistical analysis

Statistical analysis of data was performed using IBM-SPSS version 25.0 (IBM, Armonk, USA). One-way analysis of variance (ANOVA) was applied to determine the statistical significance of the differences in the volatile composition of microalgae and cyanobacteria individual species. Levene’s test was used to verify data homoscedasticity. Tukey’s test was used for pairwise comparison among individual species. When a variable was not homoscedastic, the robust Welch test was applied, and Games-Howell test was used for pairwise comparisons. In case of lack of normality of the variables for both individual RA and percentage of RA of chemical families, the non-parametric Kruskal–Wallis H test was applied. Statistical significance was declared at *P* ≤ 0.05.

## Conclusions

Microalgae and cyanobacteria are rich in VOCs and their characteristic volatile profile is strongly strain-dependent. Prokaryotic cyanobacteria, generally included within the term microalgae, are less prone to generate acyclic diketones which have been related to fishy odour notes when compared with eukaryotic microalgae. However, the influence of the individual species on the volatile profile was very significant since *AP* and *SY* cyanobacteria species showed completely different volatile profiles. Sulfur compounds can be considered as characteristic volatile compounds in marine microalgae, whereas some freshwater species such as *AP* and *SA* are rich in branched hydrocarbons. Results presented herein indicate that the volatile profile of microalgae should be individually evaluated since this profile is strongly strain-dependent. Further research is needed to relate the volatile profile with the biochemical and other compositional characteristics of each specie. Assessing the organoleptic attributes of microalgae (and cyanobacteria) is important when used for food applications, as the marine flavour and odour attributed to many microalgae strains could be used as a strategy to potentiate culinary preparations or develop novel innovative foods.

## Supplementary Information

Supplementary Information.
