# Supplementary material for: Characterisation of the volatile profile of microalgae and cyanobacteria using solid-phase microextraction followed by gas chromatography coupled to mass spectrometry
Source: Sci Rep. 2022 Mar 7;12:3661. doi: 10.1038/s41598-022-07677-4 (PMC8901680; doi:10.1038/s41598-022-07677-4)

**Supplementary Table S1.** Odour descriptors for volatile compounds detected in *Isochrysis galbana (IG)*, *Nannochloropsis gaditana (NG), Tetraselmis* sp. *(TS),* *Scenedesmus almeriensis (SA)*, *Chlorella vulgaris (CV) Synechococcus* sp (*SY*) and *Arthrospira platensis* (*AP*).

| Compounds | Odour family | Descriptors† |
| --- | --- | --- |
|  |  |  |
| Pyrazine, 2-ethyl-3,5-dimethyl- | Nutty | Burnt, almond, roasted, nutty, coffee, peanut |
| *β*-Ionone | Floral | Floral, woody, sweet, fruity, beeswax, berry, tropical |
| Butanal, 3-methyl- | Aldehydic | Ethereal, chocolate, peach, fatty |
| Methyl sulfide | Sulfurous | Sulfurous, onion, fishy, seafood, cabbage, |
| 1-Octen-3-ol | Earthy | Mushroom, earthy, green, oily, chicken, fungal, raw *Mushroom, grass, fatty |
| Propanal, 2-methyl- | Aldehydic | Fresh, floral, pungent |
| Butanal | Chocolate | Pungent, cocoa, musty, green, malty, bready |
| Furan, 2-ethyl- | Chemical | Chemical, sweet, burnt, earthy, malty, *rubber, pungent, musty |
| Heptanal | Green | Fresh, fatty, green, herbal, cognac, |
| 2-Octenal, (*E*)- | Fatty | Fresh, cucumber, fatty, green, banana, herbal, waxy, green, leafy, oxidised, oil like |
| B-Cyclocitral | Tropical | Tropical, saffron, herbal, clean, sweet, rose, tobacco, green, fruity |
| 4-Heptenal, (*Z*)- | Green | Oily, fatty, green, dairy, milky, creamy, *biscuit, creamy, fatty |
| *α*-Ionone | Floral |  |
| Hexanal | Green | Fresh, green, fatty, grassy, leafy, fruity, sweaty, woody |
| Ethyl acetate | Ethereal | Ethereal, fruity, sweet, weedy, green, grape |
| 1-Hexanol | Herbal | Ethereal, fusel, oily, fruity, alcoholic, sweet, green *Green, grassy, fatty, leafy |
| Furan, 2-pentyl- | Fruity | Fruity, green, earthy, beany, vegetable, metallic, *green, bean like, pungent |
| 2-Heptanone, 6-methyl- | Camphoreous |  |
| Propanal | Ethereal | Ethereal, pungent, earthy, winey, cognac, whiskey, brandy, cocoa, nutty, meaty, grape, *solvent, pungent |
| 2,4-Heptadienal, (*E,E*)- | Fatty | Fatty, green, oily, vegetable, cinnamon |
| Pyrazine, 2-ethyl-5-methyl- | Coffee | Coffee, beany, nutty, grassy, roasted |
| 1-Penten-3-one | Spicy | Pungent, peppery, mustard, ethereal, garlic, onion, |
| 2,3-Pentanedione | Buttery | Pungent, sweet, buttery, creamy, nutty, cheesy |
| 2-Octanone | Earthy | Earthy, weedy, natural, woody, herbal, *soapy, floral, musty, cheesy |
| Benzene, ethenyl- | Balsamic | Sweet, balsamic, floral, plastic |
| 5-Hepten-2-one, 6-methyl | Citrus | Citrus, green, musty, lemongrass, apple, creamy, cheesy, banana, *sweet, fruity |
| 2-Hexenal, (*E*)- |  | Stink bug, bitter, almond |
| 2-Penten-1-ol, (*E*)- | Green | Green, plastic, rubber |
| 2,4-Heptadienal, (*E,Z*)- |  | Fried, fatty, nutty |
| 3,5-Octadien-2-one (*E,E*)- | Fruity | Fruity, green, grassy, *woody, mushroom, hay, fresh |
| Cyclohexanone, 2,2,6-trimethyl- | Thujonic | Pungent, labdanum, honey, cistus, camphoreous |
| 2-Heptanone | Cheesy | Fruity, spicy, creamy, ketonic, sweet, herbal, coconut, woody, cheese, green, banana |
| 1-Pentanol | Fermented | Pungent, fermented, bready, yeasty, fusel, winey, solvent-like |
| Furan, 2-methyl- | Chocolate | Ethereal, acetone, chocolate |
| Isophorone | Woody | Cooling, woody, sweet, green, fruity, camphoreous, leathery, musty, cedarwood, tobacco |
| 2-Pentenal, 2-methyl- | Fruity | Pungent, fruity, juicy, ripe, |
| 2-Hexen-1-ol, (*Z*)- | Green | Walnut, medicinal, green, leaf, green bean, soapy, narcissus, whiskey |
| Pyrazine-2,6-dimethyl- | Chocolate | Cocoa, roasted, nutty, meaty, roasted, meaty, coffee, *roasted nuts, fried potato |
| 2-Butanol, 3-methyl- | Fruity | Musty, alcoholic, fusel, vegetable, cider cocoa, cheesy |
| 1-Butanol | Fermented | Fusel, oily, sweet, balsamic, whiskey |
| 2-Butenal, 2-methyl- | Green | Pungent, green, ethereal, nutty, fruity |
| Butanoic acid, 3-methyl- | Cheesy | Sour, sweaty, cheesy, tropical, cheesy, dairy, acidic, pungent, fruity ripe, fatty |
| 2-Penten-1-ol, (*Z*)- | Green | Green, phenolic, ethereal, metallic, cherry, narcissus, fruity |
| Benzaldehyde | Fruity | Sharp, sweet, bitter, almond, cherry, *bitter almond, woody, burnt |
| 2-Pentenal, (*E*)- | Green | Pungent, green, fruity, apple, orange, tomato |
| 3-Penten-2-one | Fruity | Acetone, fruity, phenolic, fishy |
| 1-Penten-3-ol | Green | Pungent, horseradish-like, green vegetable, tropical fruity nuances |
| Methyl acetate | Ethereal | Ethereal, solvent, fruity, winey, cognac, rummy |
| Pyrazine, 2,5-dimethyl- | Chocolate | Cocoa, roasted, nutty, beefy, roasted, beefy, woody, grassy, |
| Benzene, ethyl- | Ethereal | Ethereal, floral, sweet |
| Pentanal | Fermented | Fermented, bready, fruity, nutty, berry |
| Octane | Gasoline |  |
| Nonane | Gasoline |  |
| Decane |  |  |
| Undecane |  |  |
| Pyrazine, methyl- | Nutty | Nutty, cocoa, roasted, chocolate, musty, peanut green, earthy, *fishy, nutty, ammoniacal |
| 2-Butanone | Ethereal | Acetone, ethereal, fruity, *ethereal, cheese, chemical |
| 2-Propanone | Solvent | Solvent, ethereal, apple, pear, *glue, fruity |
| Heptane | Ethereal | Sweet, ethereal |
| 2-Pentanone | Fruity | Sweet, fruity, ethereal, winey, banana, woody, fermented, *sweet, fruity, |
| Safranal | Herbal | Fresh, herbal, phenolic, metallic, spicy, rosemary, tobacco, woody, camphoreous, powdery |
| Menthyl acetate | Mentholic | Tea, cooling, minty, fruity, berry |
| 3-Octen-2-one | Earthy | Earthy, spicy, herbal, sweet, mushroom, hay, blueberry, *fatty, spicy |
| 3,5-Octadien-2-one | Fatty | Fruity, fatty, mushroom |
| 4-Oxoisophorone | Musty | Musty, woody, sweet, tea, leafy, tobacco |
| Pyridine, 2-ethyl- | Green | green, grassy |
| Dimethyl sulfoxide | Alliaceous | Fatty, oily, garlic, mushroom |
| Dimethyl sulfone |  | Sulfurous, burnt |
| β-Ionone, 5,6-epoxy- | Fruity | Fruity, sweet, berry, woody, powdery, violet |

† All descriptors and odour types were obtained from <http://www.thegoodscentscompany.com/>, except descriptors labelled with an * witch were obtained from Giri et al. (2010).

**Supplementary data. Chemicals for volatile analysis**

Chemicals were supplied by Sigma-Aldrich (Madrid, Spain): cyclohexanone (≥ 99.5 %), n-hexane (≥ 95 %), 1-butanol (99.9%) 1-penten-3-ol (99 %), 2-butanol, 3-methyl- (≥ 99 %), 1-pentanol (≥ 99 %), 1-hexanol, (98%), 1-octen-3-ol (98 %), butanal (97 %), butanal, 3-methyl- (97 %), propanal (97 %), propanal, 2-methyl- (≥ 99%), hexanal (98 %), heptanal (≥ 95 %), 2-hexenal, (*E*)- (≥ 95 %), benzaldehyde (≥ 98 %), β-cyclocitral (90 %), safranal (90 %), methyl acetate (≥ 99 %), ethyl acetate (≥ 99.5 %), furan, 2-methyl- (99 %), furan, 2-ethyl- (≥ 98 %), benzene, methyl- anhydrous (99.8 %), benzene, ethenyl- (certified reference material, 200 μg/mL in methanol), heptane, 2,3-dimethyl- (98 %), 2,3-pentanedione (≥ 96 %), 2-propanone (≥ 99.9 %), 2-butanone (≥ 99 %), 2-pentanone (99 %), 2-heptanone (≥ 99 %), 2-octanone (≥ 98 %), *α*-ionone (95 %), *β*-ionone (90 %), *β*-ionone epoxide (90 %), cyclotrisiloxane, hexamethyl- (98 %), trichloromethane (≥ 99.8 %), dimethyl disulfide (≥ 98 %,), dimethyl sulfone (98 %), and saturated alkanes standard certified reference material (49452-u, C7-C40, 1000 µg/mL each component in *n*-hexane). 2-Penten-1-ol, (*E*)- (> 95 %), 2-penten-1-ol, (*Z*)- (>95 %), 2-hexen-1-ol, (*Z*)- (>93%), 2-pentenal, 2-methyl- (> 97 %), 2,4-heptadienal, (*E,E*)- (> 90 %), heptane, 2,4-dimethyl- (> 98 %), cyclohexane, methyl- (> 99 %), cyclohexane, 1,2-dimethyl- (*cis*- and *trans*- mixture, > 98 %), cyclohexane, ethyl- (> 98 %), 2-heptanone, 6-methyl- (> 98 %), 3-octen-2-one (> 96 %) and pyrazine, 2,6-dimethyl- (> 98 %) were supplied by TCI Europe Chemicals (Zwijndrecht, Belgium).

**Supplementary Figure S1.** Raw gas chromatography-mass spectrometry chromatograms of the selected microalgae and cyanobacteria strains: *Isochrysis galbana* (a), *Nannochloropsis gaditana* (b), *Tetraselmis sp. (c),* *Scenedesmus almeriensis* (d)*, Chlorella vulgaris* (e), *Synechococcus* sp. (f) and *Arthrospira platensis* (g)*.* Abundance in area arbitrary units. Time in minutes. Retention time of the internal standard, 25.50 min.

(a)


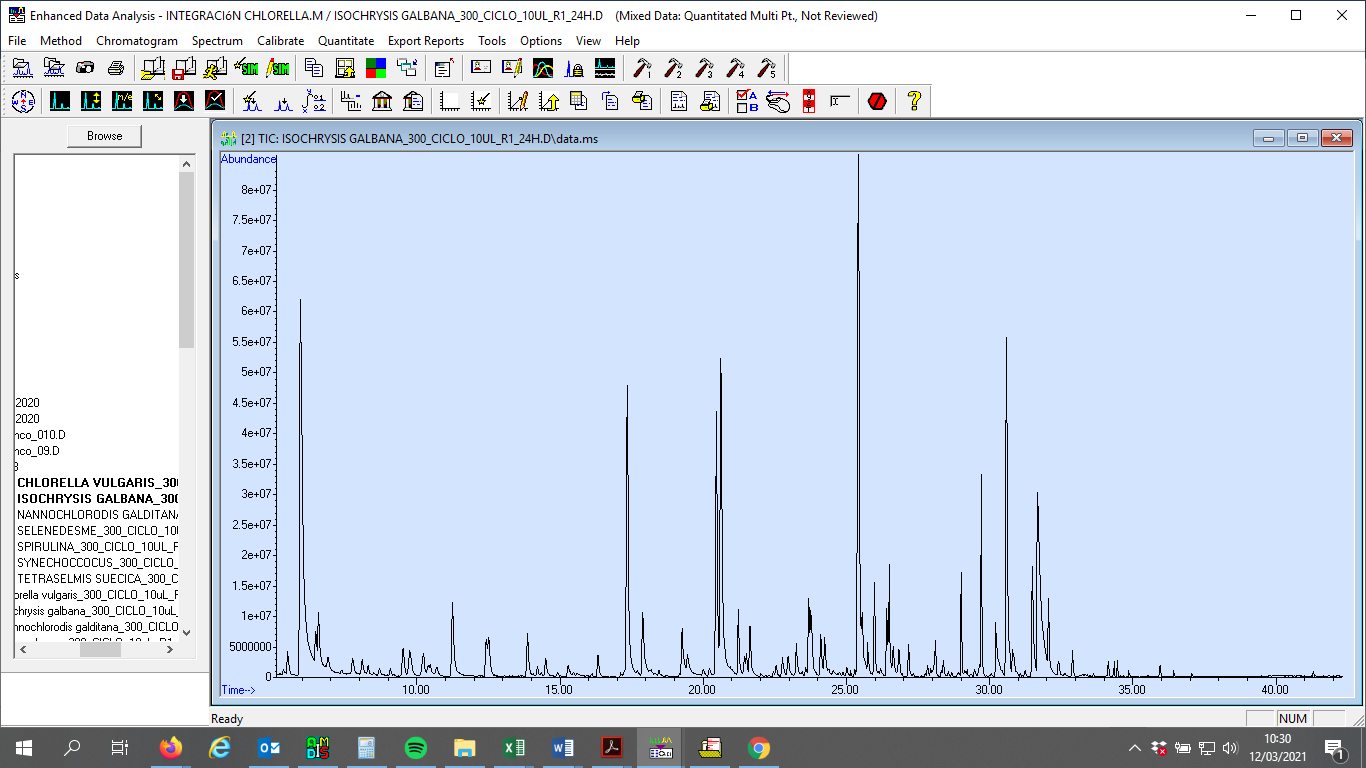


(b)


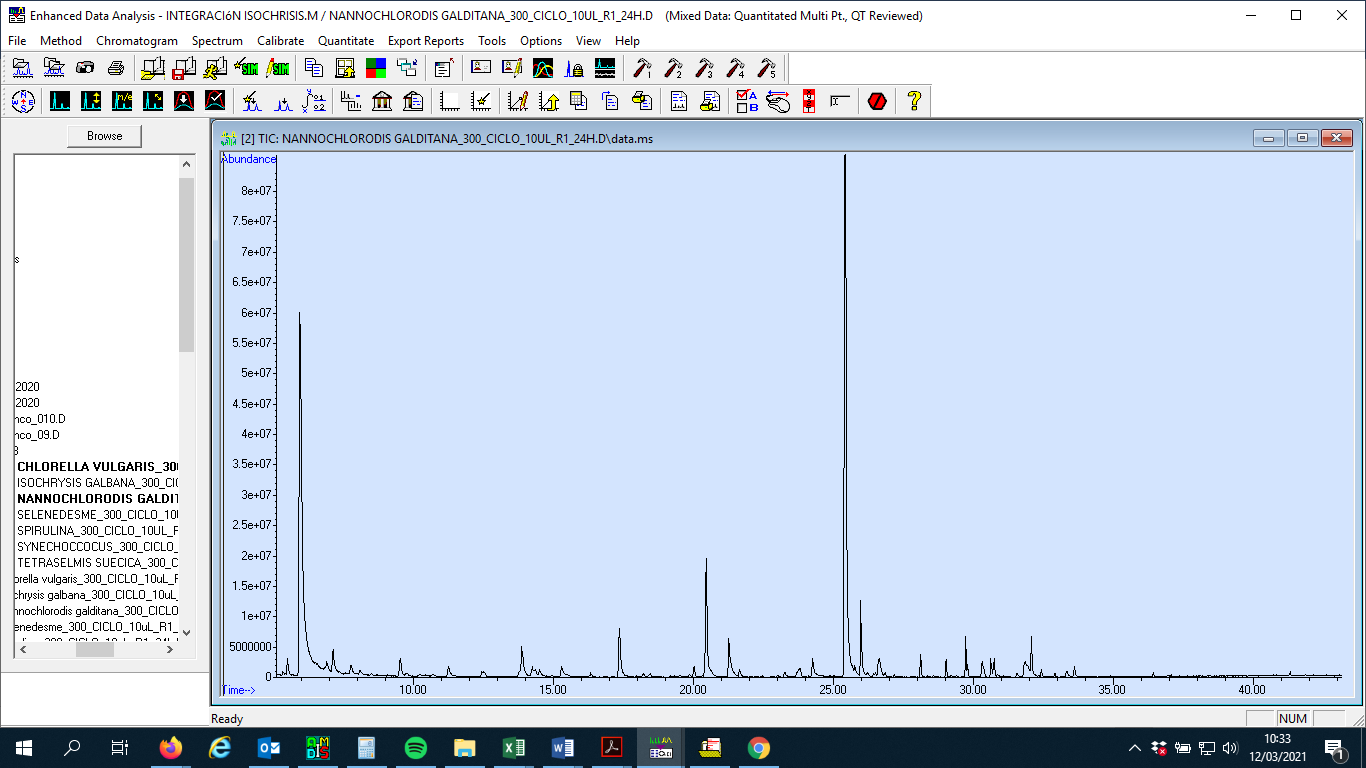


(c)


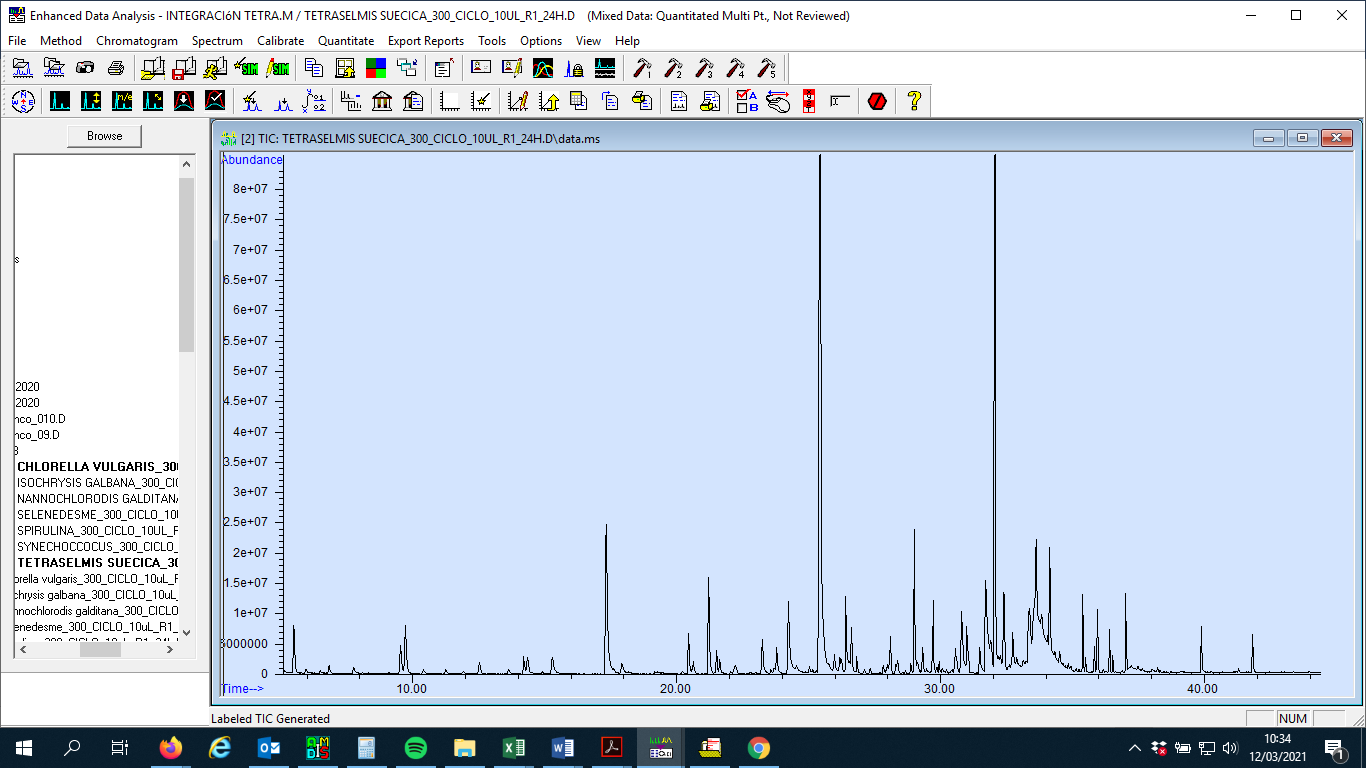


(d)


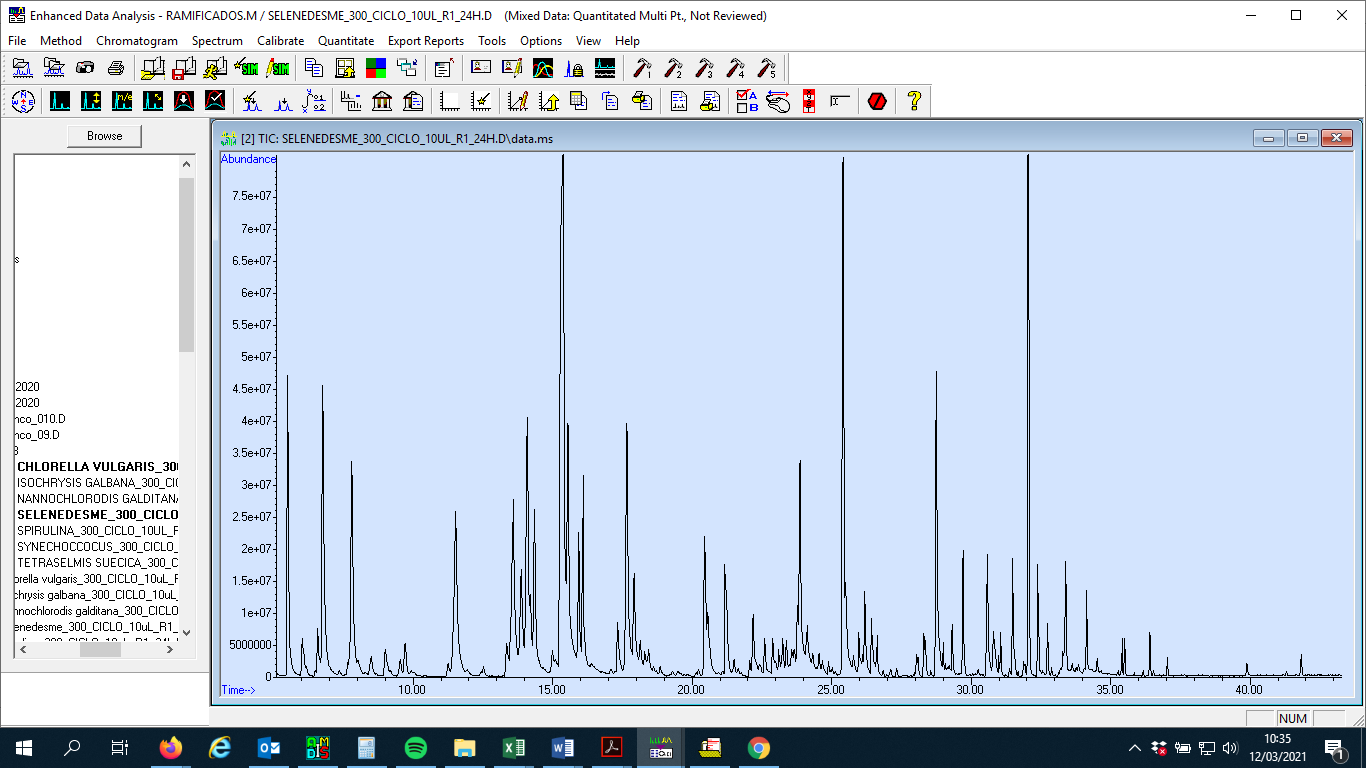


(e)


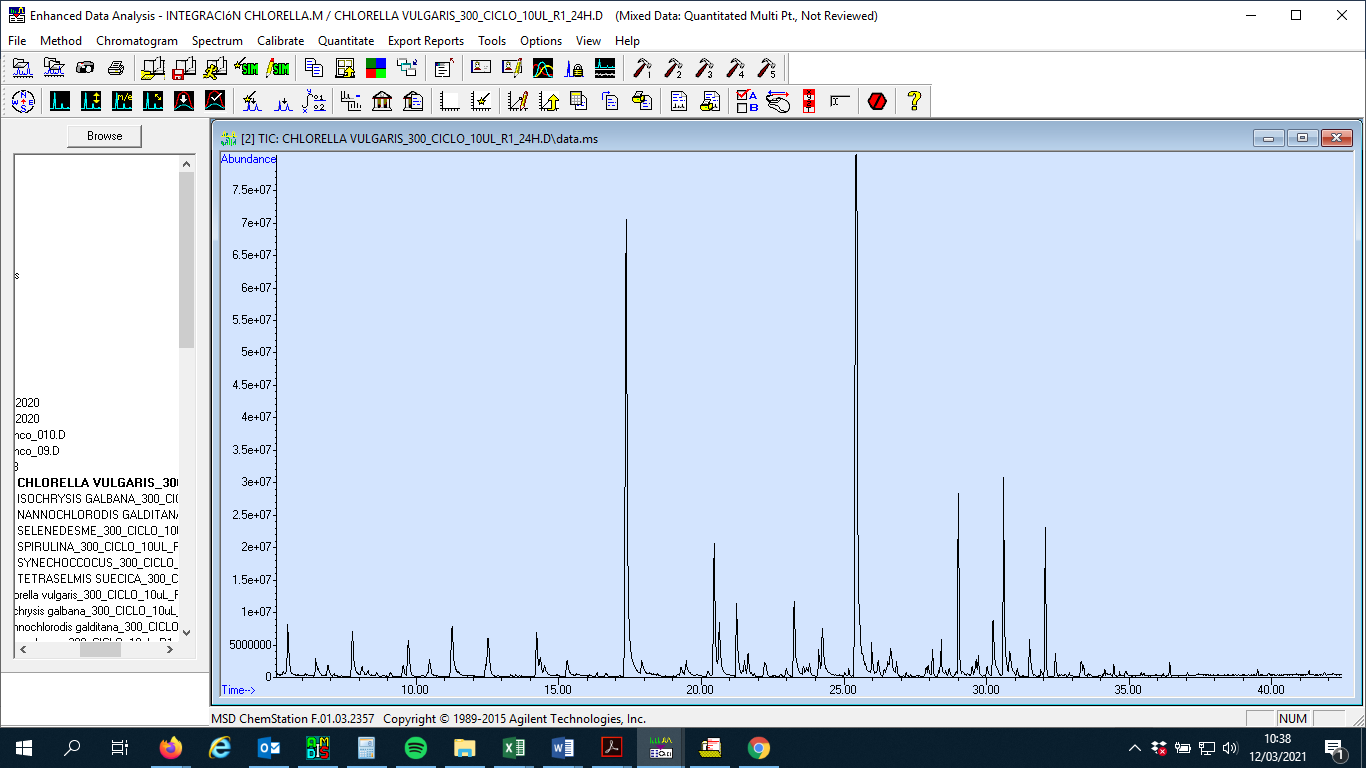


(f)


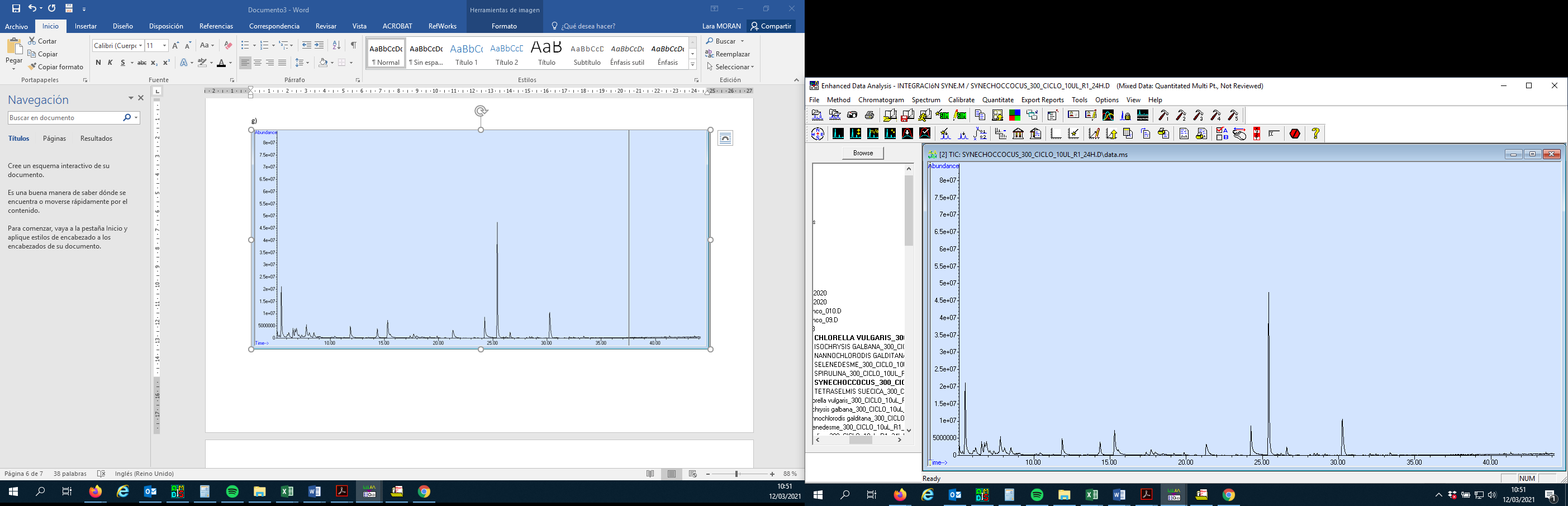


(g)


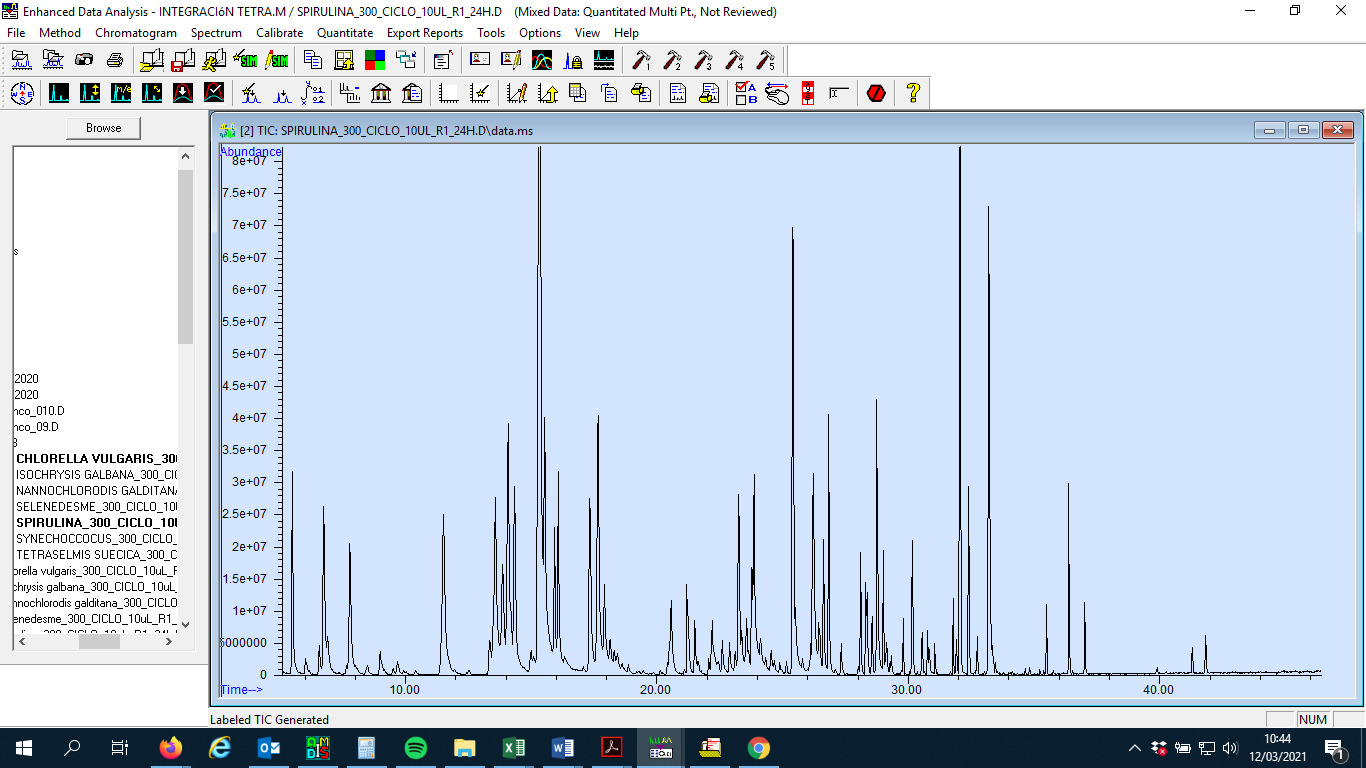

Supplement: Supplementary file 1 — Supplementary Information. [file 41598_2022_7677_MOESM1_ESM.docx]
